# Supplementary material for: Empirical methods for controlling false positives and estimating confidence in ChIP-Seq peaks
Source: BMC Bioinformatics. 2008 Dec 5;9:523. doi: 10.1186/1471-2105-9-523 (PMC2628906; doi:10.1186/1471-2105-9-523)
Supplement: Additional File 1 — A variety of html documents from the USeq web site detailing the available applications, their best usage, output file type descriptions, command line menus, etc. [file 1471-2105-9-523-S1.zip › USeqUserGuides/JavaDoc.zip!/JavaDoc/trans/graphics/package-summary.html]

trans.graphics


|  |  |  |
| --- | --- | --- |
| **Overview** \| **Package** \| Class \| **Use** \| **Tree** \| **Deprecated** \| **Index** \| **Help** | |  |
| **PREV PACKAGE**   **NEXT PACKAGE** | **FRAMES**    **NO FRAMES**     **All Classes** |  |


---

## Package trans.graphics

For graphically representing '.cel' files and intervals.

**See:**
  
          **Description**

| **Class Summary** | |
| --- | --- |
| **AggregatePlots** | For each region finds the underlying scores from the chromosome specific bar files. |
| **Box** | Box for `IntervalPlotter`. |
| **CelMasker** | Application to draw and mask a raw text cel file. |
| **CombineXYSeries** |  |
| **GenomicRegion** |  |
| **GenomicRegionGlyph** |  |
| **Graph** | Graph for `IntervalPlotter`. |
| **IntervalDrawFrame** | Frame for `IntervalPlotter`. |
| **IntervalDrawPanel** | Panel with the meat for `IntervalPlotter` |
| **IntervalPlotter** | Application to draw graphical representations of loaded Intervals. |
| **PlotFloatArrays** |  |
| **RankedSetAnalysis** | Performs set analysis on genomic regions with a visual display. |
| **RankedSetDrawFrame** | Frame for `RankedSetAnalysis`. |
| **RankedSetDrawPanel** | Panel with the meat for `RankedSetAnalysis` |
| **VirtualCel** | Builds virtual chips from float[][] files and saves each as a PNG image file. |
| **VirtualCelFrame** | Frame for `VirtualCel`. |
| **VirtualCelPanel** | Panel and calcs for `VirtualCel`. |

## Package trans.graphics Description

For graphically representing '.cel' files and intervals.

---


|  |  |  |
| --- | --- | --- |
| **Overview** \| **Package** \| Class \| **Use** \| **Tree** \| **Deprecated** \| **Index** \| **Help** | |  |
| **PREV PACKAGE**   **NEXT PACKAGE** | **FRAMES**    **NO FRAMES**     **All Classes** |  |


---
